# The small compound Icerguastat reduces muscle defects in oculopharyngeal muscular dystrophy through the PERK pathway of the unfolded protein response

**DOI:** 10.1098/rsob.230008

**Published:** 2023-04-12

**Authors:** Rima Naït-Saïdi, Aymeric Chartier, Emmanuelle Abgueguen, Philippe Guédat, Martine Simonelig

**Affiliations:** ^1^ Institute of Human Genetics, University of Montpellier, CNRS, Montpellier, France; ^2^ InFlectis BioScience, Nantes, France

**Keywords:** *Drosophila* model, Icerguastat/IFB-088, OPMD, unfolded protein response, GADD34, PEK/PERK

## Abstract

Oculopharyngeal muscular dystrophy (OPMD) is an autosomal dominant disease characterized by the progressive degeneration of specific muscles. OPMD is due to a mutation in the gene encoding poly(A) binding protein nuclear 1 (PABPN1) leading to a stretch of 11 to 18 alanines at N-terminus of the protein, instead of 10 alanines in the normal protein. This alanine tract extension induces the misfolding and aggregation of PABPN1 in muscle nuclei. Here, using *Drosophila* OPMD models, we show that the unfolded protein response (UPR) is activated in OPMD upon endoplasmic reticulum stress. Mutations in components of the PERK branch of the UPR reduce muscle degeneration and PABPN1 aggregation characteristic of the disease. We show that oral treatment of OPMD flies with Icerguastat (previously IFB-088), a Guanabenz acetate derivative that shows lower side effects, also decreases muscle degeneration and PABPN1 aggregation. Furthermore, the positive effect of Icerguastat depends on GADD34, a key component of the phosphatase complex in the PERK branch of the UPR. This study reveals a major contribution of the ER stress in OPMD pathogenesis and provides a proof-of-concept for Icerguastat interest in future pharmacological treatments of OPMD.

## Graphical abstract

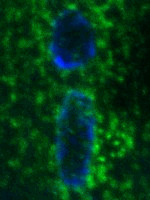

## 1. Introduction

Oculopharyngeal muscular dystrophy (OPMD) is an autosomal dominant disease that appears in the fifth decade. The disease is characterized by the progressive degeneration of specific muscles leading to ptosis (eyelid drooping), dysphagia (swallowing difficulties) and proximal limb weakness [1–3]. OPMD is caused by an expansion of GCG repeat in the gene encoding the poly(A) binding protein nuclear 1 (PABPN1) [4]. The mutation leads to a stretch of 11 to 18 alanines at the N-terminus of the protein, instead of 10 alanines in normal PABPN1. This extension induces misfolding and aggregation of PABPN1 in up to 10% of nuclei in affected muscles [5]. Aggregates contain the insoluble mutated-PABPN1, ubiquitin-proteasome system proteins, chaperones, mRNAs and RNA binding proteins [6–13].

PABPN1 has multiple functions in RNA metabolism. PABPN1 was discovered through its role in pre-mRNA cleavage and polyadenylation [14]. In this reaction, PABPN1 stimulates poly(A) polymerase activity and controls poly(A) tail lengths [14–18]. PABPN1 was then shown to be involved in alternative polyadenylation where it prevents utilization of weak proximal poly(A) sites by binding to non-canonical poly(A) signals [19,20]. PABPN1 also plays a role in polyadenylation-dependent decay or processing of nuclear RNAs by the exosome, including small nucleolar RNAs, long non-coding RNAs and abnormal mRNAs retained in the nucleus [21–25].

Defects in any of these molecular functions could participate in OPMD. Indeed, PABPN1 aggregates were reported to sequester the normal protein, leading to reduced levels of soluble PABPN1 that would contribute to OPMD pathogenesis through a loss-of-function mechanism [20,26,27]. In addition, PABPN1 aggregates or the process of aggregation are likely to substantially contribute to the pathology since decreasing the aggregation load, for example following oral treatments with small compounds, improve muscle function in *Drosophila* and mouse models of OPMD [28–32]. In line with this, it was recently shown using a *Drosophila* OPMD model that increased proteasome activity arises during OPMD progression, potentially in part through proteasome plugging with mutant PABPN1 oligomers, leading to muscle atrophy by degradation of myofibrillar proteins [33]. Thus, protein homeostasis appears to play a key role in OPMD.

**Figure 1.**
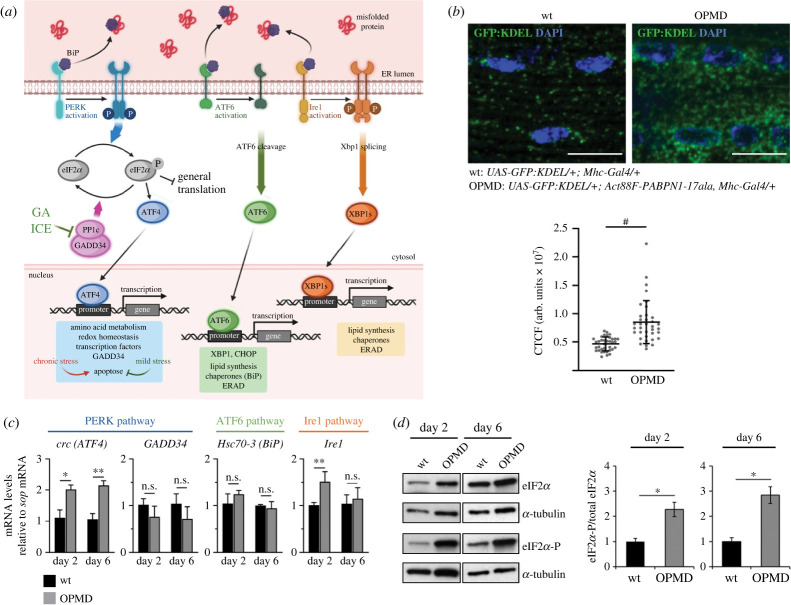
The UPR is activated in the OPMD *Drosophila* model. (*a*) Schematic representation of UPR pathway in *Drosophila* where the three branches are represented. (*b*) Immunostaining of wild-type and OPMD (*Act88F-PABPN1-17ala/+*) GFP:KDEL-expressing thoracic muscles with anti-GFP antibody. DNA was revealed with DAPI. The quantification of GFP:KDEL fluorescence was performed using imageJ and represented as the CTCF (corrected total cell fluorescence) in arbitrary units. Quantifications were from 6 wild-type muscles (*n* = 36) and 12 OPMD muscles (*n* = 36), from three biological replicates. The bars represent the means with standard deviations. ^#^*p* < 0.0001, using the unpaired-*t* test. Scale bars: 10 µm. (*c*) Quantification of UPR mRNA levels in wild-type and OPMD (*Act88F-PABPN1-17ala/+*) thoraxes at day 2 and day 6, using RT-qPCR. mRNA levels were normalized to *sop* mRNA and set to 1 in the wild-type. Means are from three to five biological replicates quantified in triplicates, error bars represent standard deviations. (*d*) Western blots of protein extracts from wild-type and OPMD (*Act88F-PABPN1-17ala/+*) thoraxes revealed with anti-eIF2*α* and anti-phosphorylated eIF2*α*. α-Tubulin was used as a loading control. Quantification of the ratio phosphorylated eIF2*α*/total eIF2*α* is shown. Means are from four biological replicates, error bars represent standard deviations. (*c, d*) *p < 0.05, **p < 0.01, n.s.: non-significant, using the unpaired-*t* test.

It has been established that reduced mitochondrial activity is one of the earliest defects in OPMD [27,34]. This defect results from an altered PABPN1 function in polyadenylation, which leads to shorter poly(A) tails of mRNAs encoding mitochondrial proteins involved in oxidative phosphorylation [34]. Decreased mitochondrial activity causes oxidative stress, which in turn produces oxidized proteins. These oxidized proteins can contribute to endoplasmic reticulum (ER) stress through accumulation of misfolded or unfolded proteins. The unfolded protein response (UPR) is initiated upon ER stress to restore ER homeostasis. The UPR is mediated by three transmembrane sensors: protein kinase R-like endoplasmic reticulum kinase (PERK, PEK in *Drosophila*), activating transcription factor 6 (ATF6) and inositol-requiring protein 1*α* (Ire1) [35–37]. The Binding Immunoglobulin Protein (BiP, Hsc70-3 in *Drosophila*) binds PERK, Ire1 and ATF6 in normal conditions. Upon ER stress, BiP recognizes and binds misfolded proteins and dissociates from the three sensors (figure 1*a*). PERK activation depends on its oligomerization and autophosphorylation. Following its activation, PERK phosphorylates eukaryotic translation initiation factor 2*α* (eIF2*α*). This leads to global inhibition of cap-dependent translation and translational activation of mRNAs encoding stress response proteins, such as activating transcription factor 4 (ATF4) and growth arrest and DNA damage-inducible protein (GADD34, also known as protein phosphatase 1 regulatory subunit 15A (PPP1R15A)). Translation activation depends on regulatory upstream ORFs (uORFs) present in the 5'-UTR of these mRNAs [38,39]. ATF4 translocates to the nucleus and activates the transcription of genes involved in the stress response, whereas GADD34 binds the catalytic subunit (PP1c) of the protein phosphatase 1 holoenzyme, which leads to dephosphorylation of eIF2*α* and recovery of translation, and thus ends the UPR [40–42]. In the two other branches of the UPR, activated ATF6 moves to the Golgi apparatus to be cleaved [43], while Ire1 is activated by oligomerization and autophosphorylation [44,45]. Ire1 promotes the splicing of the X-box binding protein 1 (XBP1) that translocates to the nucleus and activates genes involved in protein folding or degradation [46–48]. Cleaved ATF6 also translocates to the nucleus and together with XBP1 enhances through transcriptional activation, the production of chaperones such as BiP, to restore protein folding or increase protein degradation [47].

Among the small molecules identified using animal models to be active in OPMD, Guanabenz acetate (GA) is an FDA-approved antihypertensive drug that acts as an agonist of *α*2-adrenergic receptors. GA was shown to reduce muscle degeneration and PABPN1 aggregation upon oral treatment in a *Drosophila* model of OPMD [29]. GA was demonstrated to display an anti-aggregation activity by inhibiting the protein folding activity of the ribosome (PFAR) [49–51]. Synergistic positive effect between deletion of the ribosomal DNA locus and 6-aminophenanthridine, another anti-aggregative drug with the same mode of action as GA led to propose that both 6-aminophenanthridine and GA reduce OPMD phenotypes in the OPMD *Drosophila* model by preventing the PFAR activity of the ribosome [29]. More recently GA was found to show a positive effect in the A17 mouse model of OPMD (expression of PABPN1-17ala in skeletal muscles [32]), improving muscle strength and decreasing PABPN1 aggregation [52]. GA is also known to target the PERK branch of the UPR by inhibiting the phosphatase activity of the GADD34-PP1c phosphatase complex, through binding to GADD34 (figure 1*a*). This leads to prolonged eIF2*α* phosphorylation and translation inhibition, and extended protection of the ER by reducing the accumulation of misfolded proteins [53,54]. The ER stress is activated in muscles of the A17 OPMD mouse model and GA was shown to increase the levels of UPR markers in a cellular model of OPMD. Therefore, GA was proposed to reduce OPMD defects in the A17 mouse model through the UPR [52]. Together, data in the *Drosophila* and mouse models of OPMD suggest that the beneficial effect of GA on OPMD might involve both its effects in blocking the PFAR activity of the ribosome and extending the UPR.

Icerguastat (ICE), previously called IFB-088 and Sephin1, is a GA derivative with a similar effect on the UPR [54] (figure 1*a*), but devoid of its *α*2-adrenergic activity. As such, it has lower adverse effects and might be a better option for pharmacological treatments of proteinopathies. Indeed, no hypotensive effect of ICE was recorded during a phase 1 clinical trial on healthy volunteers (NCT03610334). Here, using an OPMD *Drosophila* model, we show that the UPR is activated in OPMD muscles. Using mutants of the PERK branch of the UPR, we functionally validate that this branch of the UPR participates in OPMD. We further show that ICE has a positive effect when provided orally to OPMD flies, in decreasing muscle weakness and reducing the PABPN1 aggregation load. ICE acts synergistically with reduced *GADD34* gene dosage, suggesting that its effect in OPMD depends on its known role in preventing eIF2*α* dephosphorylation by the GADD34–PP1c phosphatase complex. Together these data reveal the functional implication of the ER stress response in OPMD and identify ICE as a potential candidate for future pharmacological treatments of OPMD and other proteinopathies involving the ER stress response.

## 2. Results

### 2.1. The PERK pathway is activated in the *Drosophila* model of OPMD

We previously developed *Drosophila* models of OPMD by expressing mammalian alanine-expanded PABPN1 (PABPN1-17ala) in muscles. PABPN1-17ala is specifically expressed in muscles either from larval stages using the *UAS/Gal4* system and the *Myosin heavy chain-Gal4* (*Mhc-Gal4*) driver or in adult thoracic muscles using the *Act88F-PABPN1-17ala* transgene [33,34,55]. Both models lead to a number of flies with abnormal wing position resulting from defective muscle function and progressive muscle degeneration [33,34,55]. To record ER stress in the OPMD *Drosophila* model, we monitored ER changes in thoracic muscles of *Act88F-PABPN1-17ala/+* flies using the GFP:KDEL reporter protein that marks the ER lumen thanks to ER targeting and retention signals [56,57]. Immunostaining of GFP:KDEL-expressing muscles showed that GFP:KDEL labelled sites were substantially enlarged in OPMD muscles, particularly around nuclei (figure 1*b*) indicating a high level of ER reorganization consistent with ER stress.

We then used RT-qPCR to quantify mRNA expression of UPR pathway components in the *Act88F-PABPN1-17ala* model, at days 2 and 6 of adulthood. In the PERK branch, *ATF4* is known to be regulated at transcriptional level, in addition to its translational regulation by eIF2*α* [58]. *Drosophila* ATF4 is encoded by the *cryptocephal* (*crc*) gene [59]. We found that *crc* mRNA levels were higher in OPMD muscles compared to wild-type at both days 2 and 6 (figure 1*c*). By contrast, *GADD34* that is only regulated at the translation level in *Drosophila* [60] was not overexpressed in OPMD muscles using RT-qPCR (figure 1*c*). We quantified *Hsc70-3* (*BiP*) and *Ire1* mRNAs to assess activation of the ATF6 and Ire1 branches of the UPR, respectively. *Hsc70-3* is known to be transcriptionally activated by ATF6 (figure 1*a*) and *Drosophila Ire1* is also activated at transcriptional level [61]. *Hsc70-3* mRNA levels were not higher than wild-type in OPMD muscles, suggesting a lack of activation of the ATF6 pathway, whereas *Ire1* mRNA levels were higher at day 2, possibly consistent with its reported early activation under mild ER stress [62,63]. We next quantified eIF2*α* phosphorylation, a key marker of the PERK branch of the UPR, using western blots (figure 1*d*; electronic supplementary material, figure S1). eIF2*α* phosphorylation was higher in OPMD muscles compared to wild-type, at both day 2 and day 6 of adulthood.

These results reveal ER stress and activation of the PERK branch of the UPR in the OPMD *Drosophila* model.

### 2.2. PERK pathway mutants reduce muscle defects in OPMD flies

**Figure 2.**
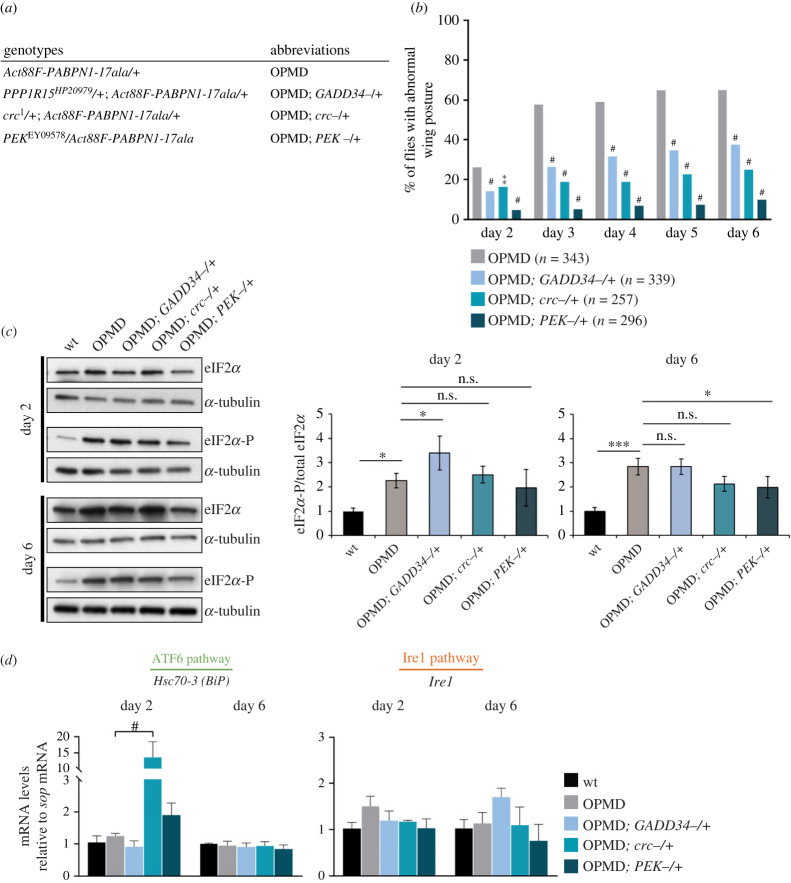
Half dosage of UPR genes reduces OPMD wing position defects. (*a*) Genotypes and abbreviations used in figures 2, 3 and 5. (*b*) Wing position defects were scored in OPMD (*Act88F-PABPN1-17ala/+*) flies in the absence or presence of UPR heterozygous mutants, each day between days 2 and 6. The numbers of scored flies are indicated (*n*). Quantifications were from of three biological replicates. **p < 0.01, ^#^*p* < 0.0001, using the Chi2-test. (*c*) Western blots of protein extracts from OPMD thoracic muscles in the absence or presence of UPR hererozygous mutants, revealed with anti-eiF2*α* and anti-phosphorylated eiF2*α*. α-Tubulin was used as a loading control. Quantification of the ratio phosphorylated eiF2*α*/total eiF2*α* is shown. Means are from four biological replicates, error bars represent standard deviations. (*d*) Quantification of UPR mRNA levels in OPMD thoracic muscles in the absence or presence of UPR hererozygous mutants, at day 2 and day 6, using RT-qPCR. mRNA levels were normalized to *sop* mRNA and set to 1 in the wild-type. Means are from three biological replicates quantified in triplicates, error bars represent standard deviations. (*c*,*d*) *p < 0.05, ***p < 0.001, ^#^*p* < 0.0001, n.s. or no significancy indicated: non-significant, using the one-way ANOVA test.

Activation of the PERK branch of the UPR has a protective role under acute ER stress through the repression of general translation. However, PERK activation is detrimental under chronic stress through its role in activating apoptosis [64,65]. We used mutants of the *Drosophila* PERK pathway, namely of *GADD34*, *crc* and *PEK* genes (electronic supplementary material, figure S2*a*) to analyse the functional role of this pathway in OPMD pathogenesis. We analysed the effect of heterozygous *GADD34*, *crc* or *PEK* mutants on the percentage of *Act88F-PABPN1-17ala* flies showing wing position defects. The *crc^1^* mutant is a known point mutant allele [66], however, *GADD34* and *PEK* mutants were uncharacterized *P*-element insertion alleles (electronic supplementary material, figure S2*a*). We, therefore, validated that the corresponding mRNA levels were reduced in these two mutants (electronic supplementary material, figure S2*b, c*). The number of flies expressing *Act88F-PABPN1-17ala* (*Act88F-PABPN1-17ala/+*) with abnormal wing posture increased from 26% to 65% between day 2 and day 6 of adulthood (figure 2*a,b*). The presence of all three heterozygous *GADD34*, *crc* or *PEK* mutants led to a significant decrease in the number of flies with abnormal wing posture (figure 2*b*). The positive effects obtained with *GADD34* and *PEK* heterozygous mutants were confirmed using *UAS-RNAi* transgenes specifically expressed in muscles with the *Mhc-Gal4* driver (electronic supplementary material, figure S2*d, e*). PEK and GADD34 have antagonist activities on eIF2*α* phosphorylation and thus translational regulation, therefore, the decrease of wing posture defects in the presence of both mutants was surprising. We analysed whether this positive phenotypic effect was linked to eIF2*α* phosphorylation by quantifying the levels of phosphorylated eIF2*α* in thoracic muscles, in the presence or absence of the three heterozygous mutants using western blots. A decrease of *GADD34* function is expected to result in increased levels of phosphorylated eIF2*α* that might reduce the ER stress through enhanced UPR response. By contrast, the levels of phosphorylated eIF2*α* are expected to decrease in the *PEK* heterozygous mutant, which might contribute to restart cap-dependent translation and increase the ER stress. It should be noted though, that variations in eIF2*α* phosphorylation levels might be difficult to record *in vivo* as quantifications were not performed under acute ER stress, but rather in flies several days old, which might blur the response. Quantification of eiF2*α* phosphorylation in thoracic muscles showed that indeed the presence of the *GADD34^−/+^* mutant in *Act88F-PABPN1-17ala/+* flies led to higher levels of phosphorylated eIF2*α* at day 2, whereas *PEK^−/+^* mutant reduced these levels with a significant decrease at day 6, and *crc^−/+^* mutant had no significant effect on eIF2*α* phosphorylation (figure 2*c*; electronic supplementary material, figure S3*a*). These results show that the level of eIF2*α* phosphorylation alone cannot explain the phenotypic improvement of OPMD flies by mutants of the PERK pathway, since reduced eIF2*α* phosphoryation in the *PEK^−/+^* mutant is not consistent with the positive effect of this mutant through the UPR. Therefore, the decrease of muscle defects by PERK pathway mutants involves different mechanisms. This is consistent with data in mouse models of neuropathy showing that reduced function of PERK or GADD34 have similar effects in improving neuronal function [67,68].

Because of the crosstalk between the three branches of the UPR, we investigated whether the ATF6 and Ire1 branches might be activated in response to reduced activity of the PERK branch in heterozygous mutants. The expression levels of *Hsc70-3* and *Ire1* mRNAs were quantified in thoracic muscles using RT-qPCR. *Hsc70-3* mRNA highly accumulated in *Act88F-PABPN1-17ala/+* thoracic muscles in the presence of the *crc^−/+^* mutant at day 2 (figure 2*d*). This increased levels of *Hsc70-3* mRNA might contribute to reduce muscle defects in *Act88F-PABPN1-17ala/+; crc^−/+^* flies through higher synthesis of the chaperone Hsc70-3/BiP. *Ire1* mRNA levels were not significantly affected in any of the three PERK pathway mutants (figure 2*d*).

These results reveal that targeting the PERK branch of the UPR strongly reduces muscle defects in the *Drosophila* OPMD model. This positive effect involves the UPR and increased eIF2*α* phosphorylation for *GADD34^−/+^* mutant. In addition, the PEK kinase acts in OPMD through a distinct mechanism.

### 2.3. Targeting the PERK pathway decreases PABPN1-17ala aggregation in *Drosophila*

**Figure 3.**
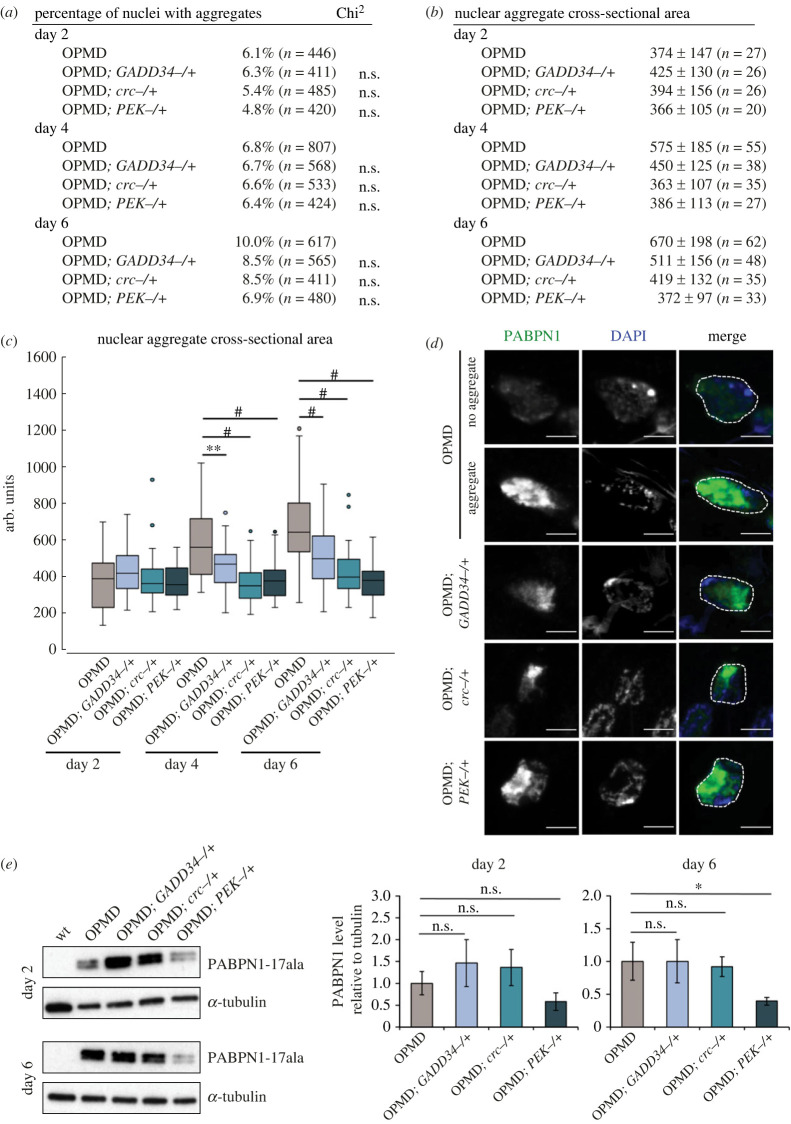
PERK pathway heterozygous mutants reduce the size of PABPN1-17ala nuclear aggregates. (*a*) Percentage of nuclei with aggregates in OPMD thoracic muscles (*Act88F-PABPN1-17ala/+*) in the absence or presence of UPR heterozygous mutants. Adult thoracic muscles were dissected at days 2, 4 and 6 and stained with anti-PABPN1 and DAPI. Nuclear aggregates were visualized and scored using both staining. n.s. = non significant, using the chi^2^ test. (*b*,*c*) Quantification of nuclear aggregate areas. Each nuclear aggregate was delimited in a focal plan and the surface area was calculated using Image J. Mean values of the surface areas are shown in arbitrary units (*b*). Distribution of nuclear aggregate surface areas are shown as boxplots (*c*). The boxes represent 50% of the values, horizontal lines correspond to the medians (50% of the values on each side of the line), and vertical bars correspond to the range. Extreme values are in open circles. **p < 0.01, ^#^*p* < 0.0001, no significancy indicated: non-significant, using the one-way ANOVA test. (*d*) Confocal images of nuclear aggregates visualized with anti-PABPN1 staining. DNA was revealed with DAPI. In OPMD individuals, nuclear aggregates are large and can occupy most of the nucleus. An example of nucleus without aggregate is shown for comparison (no aggregate). Nuclear aggregates were smaller in the presence of UPR heterozygous mutants. Nuclei are delimited with a white dotted line in the merge. Scale bars: 5 µm. (*e*) Western blots of protein extracts from OPMD thoracic muscles in the absence or presence of UPR heterozygous mutants revealed with anti-PABPN1 antibody. α-Tubulin was used as a loading control. Quantification of PABPN1 levels is shown. Means are from three biological replicates, error bars represent standard deviations. *p < 0.05, n.s.: non-significant, using the one-way ANOVA test.

Aggregation of alanine-expanded PABPN1 is a hallmark of OPMD and genetic or pharmacological improvement of muscle function often correlates with reduced aggregation load [29,31,32,69], with some exceptions [33,34]. We, therefore, asked whether the improvement of muscle function in the presence of PERK pathway heterozygous mutants was associated with variations in PABPN1-17ala nuclear aggregates. Nuclear aggregates were visualized using anti-PABPN1 and DAPI staining in *Act88F-PABPN1-17ala/+* thoracic muscles at days 2, 4 and 6 of adulthood. As previously reported [29,55], the number and size of PABPN1-17ala nuclear aggregates increased with time in OPMD muscles (figure 3*a–d*). Strikingly, in the presence of all three heterozygous *GADD34*, *crc* and *PEK* mutants, PABPN1-17ala aggregation was reduced at days 4 and 6. Although the percentage of nuclei with aggregates was not significantly reduced, the size of aggregates was reduced at days 4 and 6, with all three mutants (figure 3*a–d*). Because muscle defects and PABPN1-17ala aggregate formation are highly dependent on the levels of mutant PABPN1 [55], we quantified its levels in the absence and presence of the heterozygous mutants, in thoracic muscles using western blots. *GADD34^−/+^* and *crc^−/+^* mutants did not affect PABPN1-17ala levels, however, the *PEK^−/+^* mutant reduced its levels, with a significant decrease at day 6 (figure 3*e*; electronic supplementary material, figure S3*b*). This low level of PABPN1-17ala in the presence of the *PEK* heterozygous mutant is likely the molecular basis of the reduced muscle defects in the presence of this mutant (figure 2*b*).

Intriguingly, the decrease in the PABPN1-17ala aggregation load in the presence of the three heterozygous mutants (figure 3*c*) appears proportional to the positive effect of these mutants on the wing position phenotype (figure 2*b*), suggesting that these mutants might act primarily through PABPN1-17ala aggregation.

### 2.4. ICE is active in OPMD *Drosophila* models

**Figure 4.**
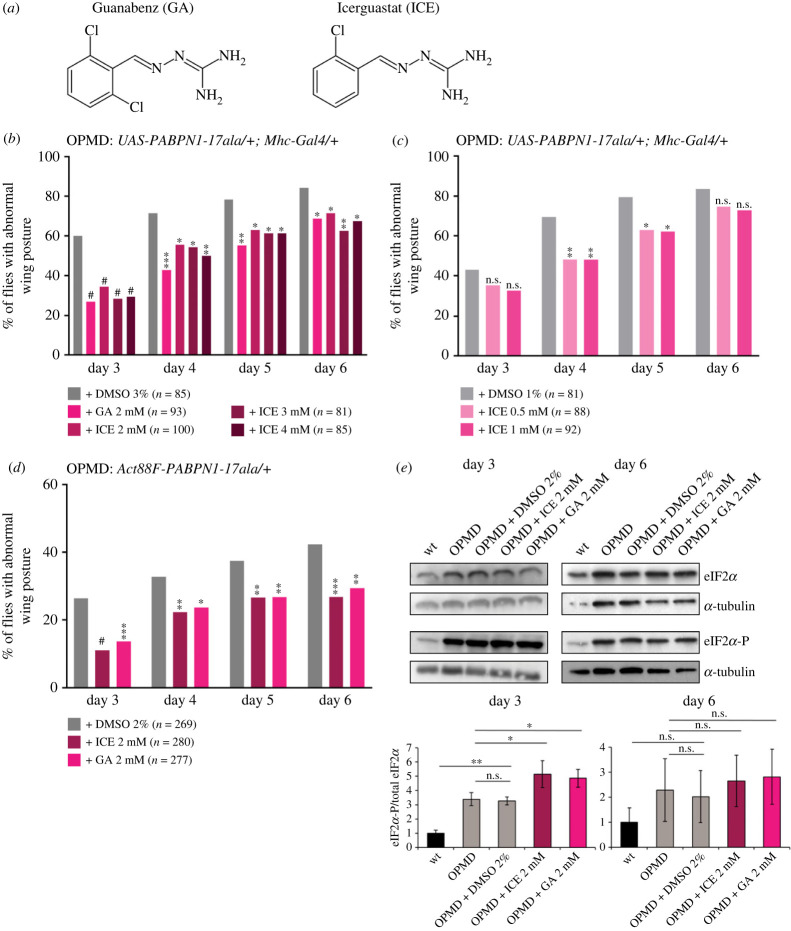
ICE reduces wing posture defects in OPMD *Drosophila* models. (*a*) Representation of GA and ICE molecules. (*b–d*) OPMD flies were transferred onto fresh instant *Drosophila* medium supplemented with ICE, GA or DMSO alone at day 2 after birth and then transferred to fresh medium with the drugs every day. Wing position defects were scored each day, between day 3 and day 6. Drug concentrations are indicated on the graphs. The numbers of scored flies are indicated (n). Quantifications were from one biological replicate in (*b*,*c*) and three biological replicates in (*d*). *p < 0.05, **p < 0.01, ***p < 0.001, ^#^*p* < 0.0001, n.s.: non-significant, using the Chi2-test. The genotypes were *UAS-PABPN1-17ala/+ ; Mhc-Gal4/+* in (*b,c*) and *Act88F-PABPN1-17ala/+* in (*d*,*e*). (*e*) Western blots of protein extracts from thoracic muscles of OPMD flies fed with the drugs or DMSO alone, revealed with anti-eiF2*α* and anti-phosphorylated eiF2*α*. α-Tubulin was used as a loading control. Quantification of the ratio phosphorylated eiF2*α*/total eiF2*α* is shown. Means are from three biological replicates, error bars represent standard deviations. *p < 0.05, **p < 0.01, n.s.: non- significant, using the one-way ANOVA test.

ICE is a GA derivative (figure 4*a*) that, as GA, was reported to prolong the UPR response by binding GADD34 and preventing eIF2*α* dephosphorylation [54]. ICE is lacking the *α*2-adrenergic activity present in GA and has therefore less side effects when used in proteinopathies. We tested the effect of ICE on two different OPMD *Drosophila* models.

First, we used the *UAS/Gal4* system to express PABPN1-17ala in muscles with the *Mhc-Gal4* driver. Flies expressing PABPN1-17ala were fed with ICE at 2 to 4 mM in the food from day 1 of adulthood and the number of flies with wing position defects were quantified at days 3 to 6. GA that has a positive effect on OPMD in *Drosophila* and mouse models [29,52] was used as a positive control. ICE had a beneficial effect in decreasing the number of OPMD flies with abnormal wing posture at the three concentrations of 2, 3 and 4 mM, compared to DMSO alone (figure 4*b*). ICE efficiency was stronger at day 3 when the wing position defects was lower, and was comparable between the three concentrations and comparable to GA efficiency at 2 mM. This indicated that the positive effect of the drug had already reached a plateau at the 2 mM concentration. We therefore analysed the effect of ICE at the lower concentrations of 1 and 0.5 mM in the food (figure 4*c*). The drug conserved a positive effect on the wing posture phenotype at these concentrations, but its efficiency decreased with a significant reduction of wing position defects at days 4 and 5 only (figure 4*c*).

Second, to confirm the positive effect of ICE on OPMD, we analysed its efficiency on the *Act88F-PABPN1-17ala* model. Similarly, *Act88F-PABPN1-17ala/+* flies were fed with 2 mM ICE in the food and wing position defects were recorded at days 3 to 6. ICE showed a positive effect in this model also, with reduced numbers of flies showing wing position defects at all time points, compared to DMSO alone (figure 4*d*). As with the *UAS-PABPN1-17ala* model, the drug efficiency was higher at day 3 and was comparable to that of GA. We then asked whether the positive effect of ICE involves its known activity in inhibiting eIF2*α* dephosphorylation. We used western blots to quantify the levels of phosphorylated eIF2*α* in thoracic muscles of *Act88F-PABPN1-17ala/+* flies fed with 2 mM ICE or GA in the food. eIF2*α* phosphorylation was higher following treatment with both ICE and GA at day 3 (figure 4*e*; electronic supplementary material, figure S4*a*). At day 6, eIF2*α* phosphorylation tended to be higher following ICE or GA treatment, but the increase was not significant (figure 4*e*; electronic supplementary material, figure S4*a*), possibly consistent with the lower efficiency of the compounds with time (figure 4*d*). These data suggest that ICE efficiency on muscle defects depends on its activity in the UPR in favouring translational repression. In addition, the same activity contributes to the beneficial effect of GA in OPMD.

We conclude that ICE is active in reducing OPMD muscle defects in *Drosophila*, with an efficiency similar to that of GA. These data also suggest that the positive effect of ICE involves its activity in targeting the PERK pathway, lengthening the UPR response.

### 2.5. ICE acts through GADD34 to reduce OPMD muscle defects

**Figure 5.**
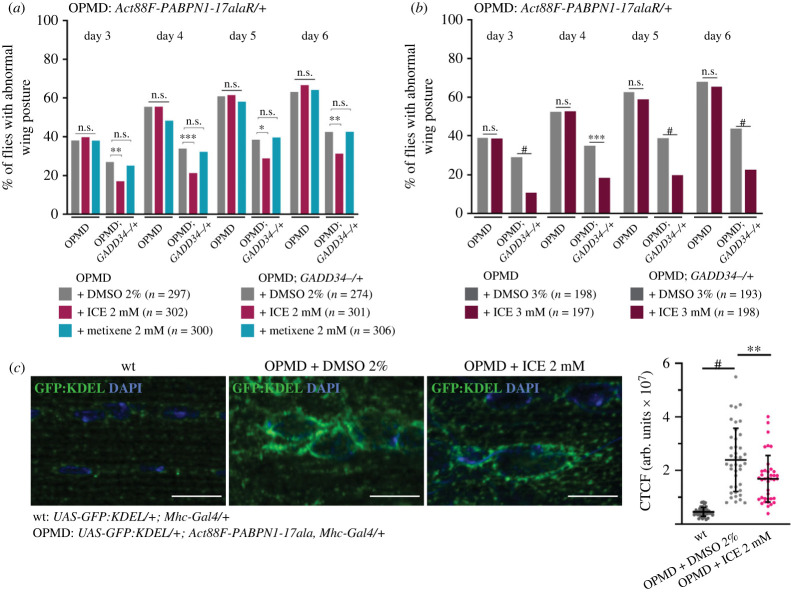
ICE acts on OPMD through its GADD34 target. (*a*,*b*) Wing position defects were scored for OPMD (*Act88F-PABPN1-17alaR/+*) and *OPMD; GADD34^−^*^/+^ flies fed with drugs or DMSO alone. Flies were transferred onto fresh instant *Drosophila* medium supplemented with ICE, metixene or DMSO alone at day 2 after birth and then transferred to fresh medium with the drugs every day. Wing position defects were scored each day, from day 3 to day 6. Drug concentrations are indicating on the graphs. The numbers of scored flies are indicated (*n*). Quantifications were from three biological replicates in (*a*) and two biological replicates in (*b*). *p < 0.05, **p < 0.01, ***p < 0.001, *^#^p* < 0.0001, n.s.: non-significant, using the Chi2-test. (*c*) Immunostaining of wild-type and OPMD (*Act88F-PABPN1-17ala/+*) GFP:KDEL-expressing thoracic muscles with anti-GFP antibody. DNA was revealed with DAPI. OPMD flies were fed with 2 mM ICE or 2% DMSO alone. The quantification of GFP:KDEL fluorescence was performed using imageJ and represented as the CTCF (corrected total cell fluorescence) in arbitrary units. Quantifications were from 9 wild-type muscles (*n* = 40), 12 OPMD muscles from flies on 2% DMSO (*n* = 41) and 13 OPMD muscles from flies on 2 mM ICE (*n* = 39), from two biological replicates. The bars represent the means with standard deviations. **p < 0.01; ^#^*p* < 0.0001, using the unpaired *t-*test. Scale bars: 10 µm.

We used the genetic approach to confirm that ICE positive phenotypic effect depended on its ability to prevent GADD34-PP1c phosphatase complex activity. The *Act88F-PABPN1-17alaR* strain contains the same transgene as the *Act88F-PABPN1-17ala* strain, but its genetic background was changed by consecutive backcrosses. The resulting *Act88F-PABPN1-17alaR/+* individuals showed a stronger OPMD phenotype, namely more individuals had wing position defects than *Act88F-PABPN1-17ala/+* (compare figure 4*d* and figure 5*a*), and importantly, they were not sensitive to oral treatment with 2 mM ICE (figure 5*a*). We took advantage of this condition where 2 mM ICE concentration was suboptimal for *Act88F-PABPN1-17alaR/+* flies to address a potential synergy with the *GADD34* heterozygous mutant. We found that the *GADD34^−/+^* mutant was able to reduce the percentage of flies with abnormal wing posture of *Act88F-PABPN1-17alaR/+* flies. When ICE at 2 mM was provided to *GADD34^−/+^; Act88F-PABPN1-17alaR/+* flies, the small molecule acted synergistically with *GADD34^−/+^* in decreasing the percentage of flies with abnormal wing posture to a higher level than *GADD34^−/+^* alone (figure 5*a*). A similar synergistic effect with *GADD34^−/+^* but more pronounced was observed when ICE was provided at 3 mM (figure 5*b*). To confirm that the observed synergy depended on a direct link between ICE and *GADD34^−/+^* mutant, we used another small molecule, metixene that is active in the OPMD *Drosophila* model but acts by reducing protein aggregation through the PFAR activity of the ribosome, not through the UPR [28]. As ICE, metixene at 2 mM was not active on *Act88F-PABPN1-17alaR/+* flies (figure 5*a*), whereas it shows positive effect on *Act88F-PABPN1-17ala/+* flies [28]. However, in contrast to ICE, it did not act synergistically with *GADD34^−/+^* as the number of *GADD34^−/+^; Act88F-PABPN1-17alaR/+* flies with abnormal wing posture did not decrease in the presence of metixene (figure 5*a*).

We, next analysed a direct effect of ICE on the ER by recording changes in the ER structure using immunostaining of muscles expressing the *GFP:KDEL* reporter. We confirmed the enlargement of the ER lumen in OPMD muscles compared to wild-type (figure 5*c*). Strikingly, GFP:KDEL staining was significantly reduced when OPMD flies were fed with food containing 2 mM ICE, compared to DMSO alone, revealing a reduction of the ER, and thus decreased ER stress in the presence of the small molecule (figure 5*c*).

Together, these results are consistent with ICE acting in OPMD through its known function in inhibiting eIF2*α* dephosphorylation by the GADD34-PP1c phosphatase complex and maintaining translation inhibition to reduce ER stress.

### 2.6. ICE reduces PABPN1-17ala aggregation in *Drosophila*

**Figure 6.**
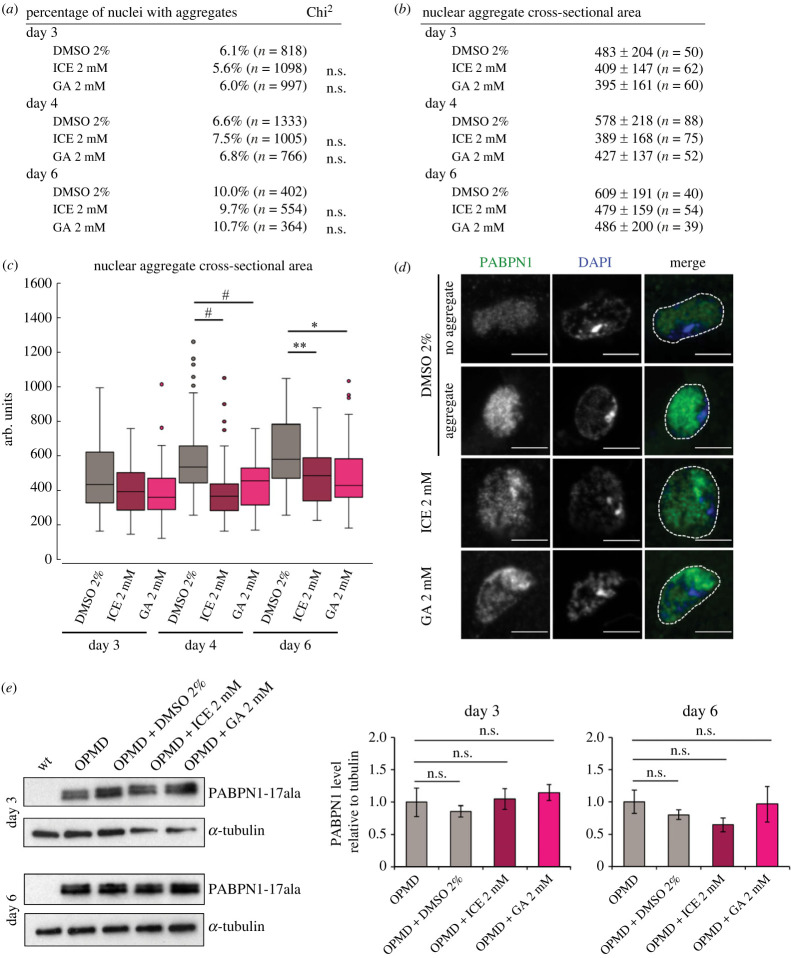
Oral treatment with ICE reduces PABPN1-17ala nuclear aggregates. (*a*) Percentage of nuclei with aggregates in OPMD thoracic muscles (*Act88F-PABPN1-17ala/+*). Adult flies were fed with the drugs that were provided in fresh medium every day from day 2. Adult thoracic muscles were dissected at days 3, 4 and 6 and stained with anti-PABPN1 and DAPI. Nuclear aggregates were visualized and scored using both staining. n.s.: non significant, using the Chi2-test. (*b*,*c*) Quantification of nuclear aggregate areas. Each nuclear aggregate was delimited in a focal plan and the surface area was calculated using ImageJ. Mean values of the surface areas are shown in arbitrary units (*b*). Distribution of nuclear aggregate surface areas are shown as boxplots (*c*). Legend is as in figure 3*b*, *c*. *p < 0.05, **p < 0.01, ^#^*p* < 0.0001, no significancy indicated: non-significant, using the one-way ANOVA test. (*d*) Confocal images of nuclear aggregates visualized with anti-PABPN1 staining. DNA was revealed with DAPI. Examples of nuclei with or without an aggregate in OPMD individuals fed with DMSO alone. Nuclear aggregates were smaller when flies were raised on drug-supplemented medium. Examples of small aggregates at day 4 in the presence of 2 mM ICE or GA. Nuclei are delimited with a white dotted line in the merge. Scale bars: 5 µm. (*e*) Western blots of protein extracts from thoracic muscle of OPMD flies fed with the drugs or DMSO alone revealed with anti-PABPN1 antibody. α-Tubulin was used as a loading control. Quantification of PABPN1 levels is shown. Means are from three biological replicates, error bars represent standard deviations. n.s.: non-significant, using the one-way ANOVA test.

The decrease of muscle defects with PERK pathway heterozygous mutants correlates with reduced PABPN1-17ala aggregation in muscle nuclei. We, therefore, analysed whether ICE oral treatment also led to reduced PABPN1-17ala aggregate formation. Similarly to GA treatment [29] (figure 6*a–d*), the number of nuclei containing aggregates was not affected following oral treatment with 2 mM ICE (figure 6*a*), but the size of aggregates was significantly reduced over time (figure 6*b–d*). This smaller size of PABPN1-17ala aggregates was not due to lower levels of PABPN1-17ala protein in thoracic muscles following ICE treatment (figure 6*e*, electronic supplementary material, figure S4*b*).

These results show that oral treatment with ICE decreases PABPN1-17ala aggregation in *Drosophila* OPMD muscles, similarly to *GADD34* heterozygous mutant. They reinforce the notion that ICE would act positively on OPMD muscles through GADD34-PP1c complex activity.

## 3. Discussion

Several molecular pathways have previously been described to participate in OPMD pathogenesis. Here, we show that the ER stress and the downstream UPR pathway play a functional role in OPMD defects using the *Drosophila* model. ER stress and activation of the UPR have been recently reported in the A17 mouse model of OPMD [52]. Here we use the genetic approach to demonstrate that modulation of the PERK branch of the UPR has a positive effect in reducing OPMD phenotypes, in part through the repression of translation.

We find that the PERK branch is the main pathway activated in the *Drosophila* model of OPMD and maintained with time. In particular, eIF2*α* phosphorylation that prevents cap-dependent translation is increased in *Drosophila* OPMD muscles. Analysis of mRNA levels indicates that the Ire1 branch also appears to be activated, however, only at early stages. The Ire1 branch has been reported to be activated upon mild ER stress and beneficial [70–73]. By contrast, activation of the PERK pathway can be beneficial or detrimental for cells depending of the type of stress [63]. Upon mild stress, activation of the PERK pathway reduces the accumulation of misfolded proteins by inhibiting global translation through eIF2*α* phosphorylation. However, during chronic stress the PERK branch can activate an apoptotic cascade followed by cell death [64,65]. Both these mechanisms downstream of the PERK branch of the UPR might modulate OPMD defects. Quantification of eIF2*α* phosphorylation shows that it increases in OPMD *Drosophila* muscles in the presence of *GADD34* heterozygous mutant, at early stages. This is consistent with a beneficial role of the PERK branch for OPMD at this early stage, through translation inhibition that would decrease the load of misfolded proteins in the ER. This beneficial effect would be enhanced by reducing the level of active GADD34-PP1c phosphatase in *GADD34* heterozygous mutant. By contrast, the positive effect of *PEK* heterozygous mutant on OPMD muscle defects, associated with the decrease of eIF2*α* phosphorylation at later stages (day 6) is not consistent with a positive role of PEK through preventing translation. Thus, PEK would also act through an additional mechanism in OPMD pathogenesis. PEK function could involve apoptosis as it is the case during chronic stress, since apoptosis has been described to participate in OPMD defects [55]. In addition, PABPN1-17ala levels are reduced in OPMD *Drosophila* muscles in the presence of *PEK* heterozygous mutant. Although the molecular mechanisms leading to this decrease remain unknown, a lower amount of PABPN1-17ala is expected to substantially contribute to the decrease of muscle defects since the strength of these defects correlates with PABPN1-17ala levels in the *Drosophila* model [55]. Despite being counterintuitive, similar positive effects resulting from targeting both PERK and GADD34 have been reported for other diseases. For example, targeting either PERK or GADD34 in a mouse model of Parkinson's disease results in protection against neuronal degeneration [74,75]. Similarly, in a mouse model of Charcot–Marie–Tooth disease, both ablation of PERK and inactivation of GADD34 lead to improvement of motor performance [67,68]. Although the mechanisms underlying the beneficial effect of PERK inactivation in these diseases have not been identified, further analyses would be of interest in the future, and might lead to identifying additional therapeutic targets.

In this study, we identify a new molecule, ICE with a positive effect when provided orally to OPMD flies. This small molecule is a GA derivative and has the same property as GA in inactivating the GADD34–PP1c phosphatase complex, which leads to the maintenance of global translation repression [53,54,76]. We find that ICE, similarly to GA, is beneficial for OPMD by increasing eIF2*α* phosphorylation levels. Moreover, our genetics data indicate that *GADD34* heterozygous mutant acts synergistically with ICE in lessening OPMD muscle defects. This provides functional evidence that ICE mechanism of action in OPMD involves GADD34-PP1c targeting. ICE was described to bind GADD34 and increase eIF2*α* phosphorylation levels from analyses in cell cultures [54]. However, more recent studies questioned this mode of action since ICE was found unable to disrupt the GADD34-PP1c complex in an *in vitro* set up [77,78]. Our data in the *Drosophila* model reinforce the conclusion that ICE can target the PERK branch of the UPR *in vivo*, although it could do so through a different mechanism than a direct interaction with GADD34. Interestingly, ICE has lower adverse effects than GA since it is devoid of the *α*2-adrenergic activity and thus has no hypotensive effect. Therefore, this small molecule has a higher potential for future development of pharmacological approaches to treat OPMD.

Intriguingly, both the genetic modulation of the PERK pathway and oral treatment of flies with ICE strongly reduces the size of PABPN1-17ala aggregates. Although reduction of muscle defects in OPMD animal models is not systematically associated with reduced PABPN1-17ala aggregation [33], this is nonetheless often the case. Indeed, treatments with diverse anti-aggregation drugs lead to both improvement of muscle function and reduction of aggregate formation [28,29,31,32,52]. These results have led to the conclusion that either the process of aggregation, or the presence of oligomeric forms of PABPN1-17ala play a key role in OPMD pathogenesis. Therefore, the decrease of PABPN1-17ala aggregation with PERK pathway heterozygous mutants or following ICE treatment might largely contribute to improving muscle function. The mechanisms underlying the reduction of PABPN1-17ala aggregation by alleviating the ER stress remain unknown. A direct link between PABPN1-17ala aggregation and the ER is unlikely since PABPN1 is a nuclear protein that does not transit through the ER. A possible hypothesis might involve the release of chaperone proteins upon reducing the ER stress; these chaperones would act in impeding PABPN1-17ala aggregation. In addition, although ICE was found to be less active as an anti-aggregation molecule [79], this activity might also contribute to reducing PABPN1-17ala aggregation as is the case for GA.

The positive effect of ICE in an animal model of OPMD along with its lower adverse effects make of this drug a promising therapeutic molecule for future treatments of OPMD. ICE was also shown to improve neuronal function in neurodegenerative diseases, such as Charcot–Marie–Tooth disease and amyotrophic lateral sclerosis [54,80]. Therefore, this molecule has strong potential for pharmacological approaches to diseases involving ER stress.

## 4. Material and methods

### 4.1. *Drosophila* stocks

*w^1118^* was used as a control. OPMD was induced either by using the transgenic line *Act88F-PABPN1-17ala* to express PABPN1-17ala in adult indirect flight muscles [33,34] or the transgenic line *UAS-PABPN1-17ala* crossed with the Gal4 driver line *Mhc-Gal4* to express PABPN1-17ala in all muscles from larval stages [55]. Mutant alleles were *PEK^EY0957^* (#17582 at Bloomington *Drosophila* Stock Center (BDSC)), *crc^1^/SM5* [66] (#266 at BDSC) and *PPP1R15^HP20979^/CyO* (#22330 at BDSC). Stocks containing *UAS-RNAi* constructs were *TRiP.GL00030-attP2* (*PEK-UAS-RNAi*, #35162 at BDSC) and *TRiP.HMS00811-attP2* (*GADD34-UAS-RNAi*, #33011 at BDSC); the *attP2* stock (#36303 at BDSC) was used as a negative control. The *UAS-KDEL:GFP* stock [56] (#9898 at BDSC) was used to visualize the ER. All crosses were performed at 25°C, except crosses between *UAS-PABPN1-17ala* and *Mhc-Gal4* that were maintained at 18°C; flies from these crosses were maintained at 18°C during the whole experiments.

### 4.2. Drug-supplemented medium and analysis of wing posture phenotype

Drug-supplemented medium was prepared with Instant *Drosophila* medium (Carolina Biological Supply Company) reconstituted with 2 ml of 1% yeast in water and supplemented with various concentrations of either Guanabenz acetate salt (Sigma, G110), metixene (a gift from Dr Cécile Voisset) or ICE (InFlectis BioScience) diluted in DMSO (Sigma, D2650). DMSO alone was used as control. Note that the concentration of 2 mM ICE in the food corresponds to an exposure of 0.23 mg kg^−1^/12 h, considering that a *Drosophila* ingests 20 nanoL h^−1^ [81]. This quantity of drug is in the range of that used for the treatment of OPMD mice with GA (1.7 mg kg^−1^ day^−1^) [52]. Adult males were grouped by twenty per vial. They were fed on drug-supplemented medium one day after birth and then changed to fresh drug-supplemented medium every day. Abnormal wing position was scored from day 3 to day 6 by direct observation through the vial after pooling five males in an empty vial every day. For mutant conditions, five males were pooled per vial containing regular *Drosophila* medium. Wing posture phenotype was scored every day from day 2 to day 6.

### 4.3. RNA extraction and RT-qPCR

Total RNA was prepared from five thoraxes per genotype using Trizol (Invitrogen) following recommendations of the manufacturer. DNA was digested using RQ1 RNase-Free DNase (Promega) for 30 min at 37°. Total RNA concentration was determined with nanodrop ND-1000 spectrophotometer. 1 µg of RNA was reverse transcribed for RT-qPCR using SuperScript III (Invitrogen) and random hexamers (Invitrogen). Quantitative PCR was performed on LightCycler LC480 (Roche) using LightCycler 480 SYBR Green I Master (Roche), and normalized with the *Drosophila sop* mRNA that encodes a ribosomal protein. Primers used are listed in table 1.

**Table 1.** List of primers used in this study.

gene	forward primer	reverse primer
*sop*	CACCCCAATAAAGTTGATAGACCT	ACCACCACGAGAGCCAAAT
*GADD34*	TCGAGGAAGACGAGGATGA	AGGCGAGCATTACGTGAACT
*crc (ATF4)*	TTCCGCTCACTGACTCAAAC	AGGGTCAAGGCTTCATCATC
*Ire1*	AAATCACAACAGGGCGACTC	GTGCGGAACCTGAACATTCT
*Hsc70-3 (BiP)*	AGGAAAAGGACAAGGAGCTG	GCGTCCGTTCTTGTACACAC
*PEK*	GAATTATCTGCGCCTTCAGC	CTCCGTGTCATTTTCGTCCT

### 4.4. Immunostaining and western blots

Dissection and immunostaining of adult indirect flight muscles (IFMs) were performed as described previously [55] with some modifications. Following dissection, thoraxes were fixed for 45 min in 4% paraformaldehyde, 1× PBS. For immunostaining, IFMs were incubated in 1× PBS, 0.1% Triton X-100 four times for 15 min, then blocked in 1× PBS, 0.1% Triton X-100, 1% BSA for 1 h at room temperature. Antibodies diluted in 1× PBS, 0.1% Triton X-100, 1% BSA were as follows: rabbit anti-PABPN1 at 1:1000 [82]; mouse anti-GFP at 1:1000 (Roche, #11814 460 001). DNA was revealed by 3 min staining with 1 µg ml^−1^ DAPI (Sigma-Aldrich) in 1× PBS, 0.1% Triton X-100. Fluorescent images were acquired with a Leica SP8 confocal microscope. Quantification of KDEL:GFP staining was performed using ImageJ and calculated with the CTCF (corrected total cell fluorescence) as follows: CTCF = integrated density – (selection area* mean fluorescence of the background), where integrated density = selection area × mean fluorescence of the selection. The area of nuclei was subtracted from the area of the selection. Surface of aggregates was measured and quantified using the ImageJ software. For western blots, protein extracts were obtained from five thoraxes per genotype crushed in 50 µl of 2× Laemmli buffer supplemented with 10% β-mercaptoethanol and boiled for 10 min at 95°. Samples were loaded onto 4–15% or 10% SDS-PAGE gels and transferred to nitrocellulose membranes. Each membrane was blocked for 2 h in 5% milk diluted in 1× PBS, 0.1% Tween 20. Primary antibody incubation was performed overnight at 4°C in 1× PBS, 0.1% Triton X-100, 1% BSA, 0.02% azide. Antibodies used were: rabbit anti-eIF2*α* at 1:1000 (Abcam, Ab26197), rabbit anti-phosphorylated eIF2*α* at 1:1000 (Cell Signaling, #3398S), rabbit anti-PABPN1 at 1:1000 [82], mouse anti-α-Tubulin at 1:5000 (Sigma, T9026).

## Data Availability

The data are provided in electronic supplementary material [83].
